# Phylodynamic applications in 21^st^ century global infectious disease research

**DOI:** 10.1186/s41256-017-0034-y

**Published:** 2017-05-08

**Authors:** Brittany D Rife, Carla Mavian, Xinguang Chen, Massimo Ciccozzi, Marco Salemi, Jae Min, Mattia CF Prosperi

**Affiliations:** 1 Emerging Pathogens Institute and Department of Pathology, Immunology and Laboratory Medicine, University of Florida, Gainesville, FL USA; 2 Department of Epidemiology, University of Florida, Gainesville, FL USA; 3 Department of Infectious, Parasitic and Immune-Mediated Diseases, Istituto Superiore di Sanità, Rome, Italy; 4 Unit of Clinical Pathology and Microbiology, University Campus Biomedico of Rome, Rome, Italy

**Keywords:** Molecular epidemiology, Phylodynamics, Phylogeography, Comparative phylogenetics, HIV, Zika, Dengue, WGS, MRSA

## Abstract

**Background:**

Phylodynamics, the study of the interaction between epidemiological and pathogen evolutionary processes within and among populations, was originally defined in the context of rapidly evolving viruses and used to characterize transmission dynamics. The concept of phylodynamics has evolved since the early 21^st^ century, extending its reach to slower-evolving pathogens, including bacteria and fungi, and to the identification of influential factors in disease spread and pathogen population dynamics.

**Results:**

The phylodynamic approach has now become a fundamental building block for the development of comparative phylogenetic tools capable of incorporating epidemiological surveillance data with molecular sequences into a single statistical framework. These innovative tools have greatly enhanced scientific investigations of the temporal and geographical origins, evolutionary history, and ecological risk factors associated with the growth and spread of viruses such as human immunodeficiency virus (HIV), Zika, and dengue and bacteria such as Methicillin-resistant *Staphylococcus aureus*.

**Conclusions:**

Capitalizing on an extensive review of the literature, we discuss the evolution of the field of infectious disease epidemiology and recent accomplishments, highlighting the advancements in phylodynamics, as well as the challenges and limitations currently facing researchers studying emerging pathogen epidemics across the globe.

**Electronic supplementary material:**

The online version of this article (doi:10.1186/s41256-017-0034-y) contains supplementary material, which is available to authorized users.

## Background

Globalization has dramatically changed the way in which pathogens spread among human populations and enter new ecosystems [1, 2]. Through migration, travel, trade, and various other channels, humans have and will continue to intentionally or unintentionally introduce new organisms into virgin ecosystems with potentially catastrophic consequences [3]. Humans are not the only culprits, however; global climate pattern changes can alter local ecosystems, creating favorable conditions for the rapid spread of previously overlooked or even undiscovered organisms among humans, giving rise to unexpected epidemics [4, 5]. Recent years have been marked by global epidemics of Ebola, dengue, and Zika, derived from pathogens previously restricted to local outbreaks [6]. According to the World Health Organization, more than one and a half billion people are currently awaiting treatment for neglected tropical diseases with similar potential for global spread, for which we have limited knowledge of etiology and treatment options [7]. This lack of knowledge further limits our ability to investigate the putative role of these pathogens in future epidemics or even pandemics.

**Fig. 1:**
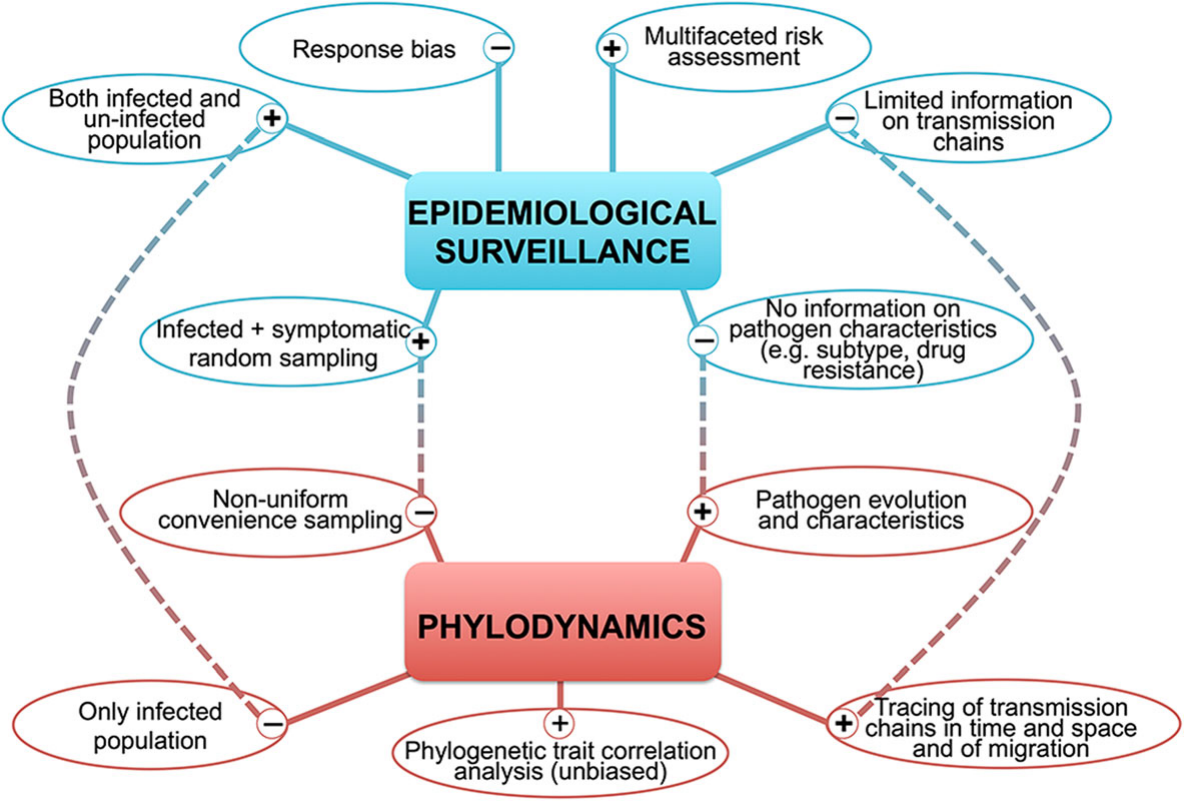
Benefits and limitations of epidemiological surveillance and phylodynamic methods employed for control of infectious disease

Epidemiological strategies have been and still are the first line of defense against an outbreak or epidemic. Despite conventionality, traditional epidemiological methods for the analysis of global infectious diseases are subject to errors from various sources (Fig. 1) and are thus often inadequate to investigate the epidemiology of an infectious disease. Putative outbreak investigations typically ensue following case notification of one of the diseases recognized by local and global public health organizations. Trained investigators subsequently collect data on cases and diagnoses to establish a disease cluster. During active surveillance, more cases may be detected through outreach to healthcare facilities and nearby health departments. Relevant case contacts, such as family, friends, and partners, are also sought to provide details on demographics, clinical diagnoses, and other potential risk factors associated with the spread of the disease [8]. However, the lack of infrastructure, trained personnel, and resources in low- and middle-income countries are prohibitive against field epidemiology investigations, as contact tracing and surveillance both require systematic, unbiased, and detailed investigations. The reconstruction and interpretation of transmission networks are often very sensitive to response, selection, and recall biases and are strictly limited by surveillance data collected in many regions with diverse socioeconomic and cultural backgrounds [9–11]. In addition, even with a highly effective surveillance system, environmental, zoonotic, and vector-borne transmission dynamics confound analysis by shadowing alternative (i.e., not human-to-human) routes of disease acquisition. Furthermore, routine analyses of pathogen subtype and drug resistance are conducted only in a subset of developed nations, wherein variation in screening assays and protocols and therapy regimens increases the discordance in surveillance [12, 13].

Despite the limitations to traditional infectious disease epidemiology, major advances in study designs and methods for epidemiological data analysis have been made over the past decade for a multifaceted investigation of the complexity of disease at both the individual and population levels [14, 15]. However, many challenges for infectious disease research remain salient in contemporary molecular epidemiology, such as the incorporation of intra- and inter-host pathogen population characteristics as influential factors of transmission. Combating current and future emerging pathogens with potential for global spread requires innovative conceptual frameworks, new analytical tools, and advanced training in broad areas of research related to infectious diseases [16–18]. An expanded multi-disciplinary approach posits advancement in infectious disease epidemiology research and control in an era of economic and health globalization [2, 16, 19, 20].

Fortunately, recent developments in phylogenetic methods have made possible the ability to detect evolutionary patterns of a pathogen over a natural timescale (months-years) and allow for researchers to assess the pathogen’s ecological history imprinted within the underlying phylogeny. When reconstructed within the coalescent framework, and assuming a clock-like rate of evolution, the evolutionary history of a pathogen can provide valuable information as to the origin and timing of major population changes [21]. Phylogenetic methods also provide key information as to the evolution of both genotypic and phenotypic characteristics, such as subtype and drug resistance (Fig. 1). Even though phylogenetic methods are also limited in certain areas, such as restriction of analysis to only the infected population, a significant subset of these limitations can be overcome by complementary use of data from surveillance (both disease and syndromic) and monitoring [22] (Fig. 1).

By integrating phylogenetic methods with traditional epidemiological methods, researchers are able to infer relationships between surveillance data and patterns in pathogen population dynamics, such as genetic diversity, selective pressure, and spatiotemporal distribution. Systematic investigation of these relationships, or phylodynamics [23], offers a unique perspective on infectious disease epidemiology, enabling researchers to better understand the impact of evolution on, for example, spatiotemporal dispersion among host populations and transmission among network contacts, and vice versa [21, 24]. The study of the interconnectedness of these pathogen characteristics was previously limited by the cost and timescale of the generation of molecular data. Recent decades have been characterized by technology with the ability to rapidly generate serial molecular data from identifiable sources for which we can obtain detailed relevant information through epidemiological surveillance, allowing for the merging of phylodynamics and epidemiology, or evolutionary epidemiology [24, 25]. Hence, progress in the field of molecular evolution has provided the opportunity for real-time assessment of the patterns associated with local, national, and global outbreaks [26], cross-species transmission events and characteristics [27], and the effectiveness of treatment strategies on current [28] and recurring epidemics [29]. These assessments are essential for monitoring outbreaks and predicting/preventing pandemic inception, a good example being the recent study of Middle East Respiratory Syndrome coronavirus global transmission [30] (Additional file 1 (Video S1)). But has the, field of evolutionary epidemiology quite reached its full potential? In this article, we systematically discuss how the application of phylodynamic methods has and will continue to impact epidemiological research and global public health to understand and control infectious diseases locally and across the globe.


Additional file 1: Video S1. Phylodynamic inference of global spread of Middle East Respiratory Syndrome coronavirus from 2008 to 2015. (MOV 14155 kb)


## The evolution of phylodynamics and overview of current methodology

In a strict sense, the concept of phylodynamics is anything but new. The phylogenetic tree reconstructed by Haeckel in 1876 using phenotypic traits [31] was used to explain the distribution of the earliest humans – the “twelve races of Man”–across the globe and the location of the “Centre of Creation.” This incorporation of both spatial information and phylogenetic relationships in the inference of population distributions and diversity among geographical locations is a branch of phylodynamics, often referred to as phylogeography. Since then, the progression of genetic sequencing technology as well as geographical information systems (GIS) has enabled evolutionary biologists to gain a higher resolution view of infectious disease dynamics.

The 21^st^ century, in particular, has witnessed unparalleled advances in methods and techniques for molecular sequence data generation and analyses. However, the relationship of progress and perfection is far from linear, along with its relationship to navigational ease. For example, phylodynamic inference has transitioned into a highly statistics-focused process with the corresponding challenges, including informative samples that can significantly affect the accuracy of results [32–34]. Several research groups [32, 33] have reviewed and/or demonstrated the impact of neglecting critical quality control steps on obtaining reliable inferences using the recently developed phylodynamic frameworks, particularly with high throughput, or next-generation, sequencing (NGS) data. Some important steps include ensuring uniform spatial and temporal sampling [32], sufficient time duration between consecutive sample collections for observing measurable evolution [33], coverage of deep sequencing, and consideration of genomic recombination [34].

The reliance on phylodynamic methods for estimating a pathogen’s population-level characteristics (e.g., effective population size) and their relationships with epidemiological data suffers from a high cost – increasing the number of inference models, and thus parameters associated with these models, requires an even greater increase in the information content, or phylogenetic resolution, of the sequence alignment and associated phenotypic data. Low coverage [35] and the presence of organism- or sequencing-mediated recombination [36], can skew estimates of the evolutionary rate and even impact the underlying tree topology, particularly when dealing with priors in the Bayesian statistical framework commonly used for phylodynamic inference. Programs such as SplitsTree [37] can take as input a nucleotide alignment and output a network in which the dual origins of recombinant sequences are displayed in a phylogenetic-like context. However, network-reconstructing programs have difficulty distinguishing actual recombination events from phylogenetic uncertainty, and branch lengths do not usually reflect true evolutionary distances [38]. Despite much work ongoing in this area, there are currently no broadly applicable methods that are able to reconstruct phylogenetic network graphs that explicitly depict recombination and allow for phylodynamic inference. Although the Bayesian framework has shown to be fairly robust with the inclusion of recombinant sequences in large population studies [39], the inclusion threshold has not been thoroughly investigated and is likely dependent on a number of factors, such as sample size and sequence length. Recombinant sequences are thus usually removed prior to analysis; however, the ability to incorporate recombinant sequences is imperative given our knowledge of the role of recombination in virus adaptation [40], for example. More details on methods that can potentially account for recombination, applicable to a variety of pathogens, are discussed by Martin, Lemey, & Posada [36].

While the traditional realm of phylogenetics has focused on rapidly evolving viruses, the development of whole-genome sequencing (WGS) has made possible the expansion of phylodynamic methods to the analysis of slower-evolving microorganisms, such as bacteria, fungi, and other cell-based pathogens. WGS has widened the range of measurably evolving pathogens, allowing for the identification of sparse, genetically variable sites, referred to as single nucleotide polymorphisms (SNPs), among populations sampled at different time points. The use of WGS in phylogenetics is highly beneficial not only in resolving relationships for slower-evolving organisms but also in reconstructing a more accurate evolutionary history (phylogeny) of an organism, rather than the genealogy (single gene), which can differ significantly from the phylogeny due to the presence of selective pressure or even genetic composition [41]. However, as with phylodynamic analysis of rapidly evolving viruses, WGS analysis of cell-based pathogens comes with its own challenges, as discussed in detail elsewhere [42].

Implementation of phylodynamic and/or phylogeographic analysis has transitioned over the last two decades from maximum likelihood to the Bayesian framework. This framework provides a more statistical approach for testing specific evolutionary hypotheses by considering the uncertainty in evolutionary and epidemiological parameter estimation. Given surveillance data (e.g., the duration of infection) and the specification of an epidemiological mathematical model, Bayesian phylogenetic reconstruction can also be used to estimate epidemiological parameters that might otherwise be difficult to quantify [21]. For example, during the early stage of an epidemic, wherein the pathogen population is growing exponentially, the rate of exponential growth can be estimated from the phylogeny using a coalescent model that describes the waiting time for individual coalescent events of evolutionary lineages. This rate estimate can be combined with knowledge of the duration of infection for a particular pathogen to estimate the basic reproduction number, *R*_0_ (e.g., [43]), as well as the prevalence of infection and number of infected hosts. Transmission dynamics can similarly be inferred following the early exponential growth of the pathogen, during which the pathogen has become endemic. Estimation of these parameters is described more thoroughly in Volz et al. [21].

With the expansion of phylodynamic methods to global epidemics, theoretical studies have found that inferences of infection dynamics within the coalescent framework are limited by the assumption of a freely mixing population [32]. This assumption is often violated with the inclusion of several isolated geographical areas with single or few pathogen introductions. Without considering this factor, population structure within a phylogeny can severely bias inferences of the evolutionary history and associated epidemiological parameters [32, 44]. To overcome this limitation, software packages such as BEAST (Bayesian Evolutionary Analysis Sampling Trees) [45–47] have recently developed algorithms that allow for the integration of coalescent, mathematical, and spatial diffusion models [48–53]. More importantly, BEAST readily implements a comparative phylogenetic approach, which incorporates parameterization of phenotypic trait evolution to identify predictors of population dynamics and spatial spread, all of which are estimated/assessed simultaneously during reconstruction of the evolutionary history [54, 55]. Statistical evaluation of the risk factors for pathogen population growth and spread can be performed concurrently with the assessment of phylogenetic resolution within the data [54], discussed above as a challenge to complex phylodynamic analyses. For example, in the absence of strong phylogenetic resolution, Bayesian statistics are more sensitive to long-branch attraction bias [56, 57], wherein rapidly evolving lineages appear to be closely related, regardless of their true evolutionary relationships. This phenomenon, therefore, influences inferences of spatiotemporal spread of the studied pathogen, as well as estimation of the relationship of pathogen population behavior with potential risk factors, such as climate change, host and/or vector distribution, accessibility and so on. The influence of low-resolution molecular data on the reliability of phylodynamic inferences highlights the importance of the implementation of the method described by Vrancken et al. [54], or even a priori estimation of the phylogenetic and temporal resolution (sufficient time between sampling) [58, 59].

Unlike other phylogenetic frameworks, Bayesian inference enables utilization of prior knowledge in the form of prior distributions (in combination with information provided by the data); however, abuse of prior knowledge is possible and can lead to incorrect conclusions. Even within the Bayesian school of thought, scientists do not always agree with regard to the specification of prior distributions under certain conditions. The incorporation of prior information is, however, intuitively appealing, as it allows one to rationalize the probability of an estimate based on previous knowledge of the typical behavior of the parameter among populations of the organism under study. But what can we do if we have no knowledge regarding a particular organism or population? This has become a more pertinent issue recently with the increasing rate of discovery, facilitated by NGS, of organisms for which we have limited prior knowledge, such as novel viruses and bacteria, [60]. One of the advantages of the Bayesian phylodynamic approach is the ability to test multiple hypotheses regarding the evolution or epidemiological models used to describe infectious disease behavior, but because of the intricate relationship of these models, reliable inferences require testing of all combinations of the individual proposed models. Although often neglected due to computational complexity, improved estimates of marginal likelihoods used for statistical model comparison have been demonstrated with less computational effort [61]. Additionally, if we know that we know nothing about the parameter in question, then, in fact, we know something. Referred to as the “objective Bayesian” approach, this ideal allows researchers to alter a normally “subjective” prior to create one that is minimally informative. This term is used because the impact of this type of prior on parameter estimation can be controlled to a minimum, allowing the data to dominate the analytical process and conclusions drawn [62]. Although similarly appealing, this approach can be particularly problematic with small datasets [63] or biased datasets, such as the exclusion of potential intermediate sampling locations [27]. The expanding volume of sequence data and increasing efforts to combine epidemiological and laboratory data in open access locations can help to improve evolutionary estimates. Additionally, the growing availability of data and collaboration can accelerate our understanding of the emergence and spread of infectious diseases through coordinated efforts by multi-disciplinary researchers across various institutions and public health organizations. More detail on the benefits of open access databases and data sharing in the context of phylogenetic epidemiology is reviewed in [64] and [65].

## Results and Discussion

### Evolutionary reconstruction of spatiotemporal transmission: dengue virus and applications to other vector-borne tropical pathogens

Combining pathogen genetic data with host population information (e.g., population density and air traffic) in a statistical framework is critical for the reliable assessment of factors potentially associated with pathogen population dynamics and geographic spread. The comparative phylogenetic approach described above [66] was used recently to identify potential determinants of the dengue virus (DENV) introduction to and spread within Brazil. Results from Nunes et al. [67] suggested that for three DENV serotypes, the establishment of new lineages in Brazil had been occurring within 7 to 10-year intervals since their primary introduction in 1985, most likely from the Caribbean. Additionally, they observed that aerial transportation of humans and/or vector mosquitoes, rather than distances between geographical locations or mosquito (particularly *Aedes aegypti*) infestation rates, were likely responsible. The study by Nunes et al. marked one of the first uses of the comparative phylogenetic approach for vector-borne tropical diseases and implies the need for a similar approach in future studies aimed at investigating transmission patterns of a broad range of emerging vector-borne viruses. For example, this approach will allow researchers to determine if specific universal factors, such as vector species, are predictive of global transmission route or if health policy and prevention strategies tailored specifically to the pathogen, irrespective of the vector, are required for effective control.

## The evolution of an epidemic revolution: Zika virus

With the development of molecular clock models for serially sampled data [68], phylogenetic analyses have helped to uncover the timing of transmission events and epidemiological origins. Moreover, when paired with comparative phylogeographic models, researchers have been able to identify risk factors most likely associated with these particular events. Since the inception of the Zika virus (ZIKV) pandemic around May of 2015 in Brazil [69], phylogeneticists and epidemiologists have sought to reveal mechanisms by which ZIKV has spread and the factors fueling the wide geographical leaps. A full-genome phylogeographic analysis of ZIKV isolates collected during 1968–2002 revealed very intricate spatiotemporal transmission patterns across Africa prior to the introduction into Asia [70]. From its origin in Uganda, two independent transmission events appeared to play a role in the spread of ZIKV from East Africa to the West circa 1920: the first involved the introduction of ZIKV to Côte d’Ivoire with subsequent spread to Senegal, and the second involved the spread of the virus from Nigeria to West Africa. Results from spatiotemporal analysis demonstrated that Uganda was the hub of the African epidemic as well as the common ancestor of the Malaysian lineages sampled during the 1966 outbreak [70].

Following the emergence and rapid spread of ZIKV in Brazil and other South American countries [69], Faria’s group sought to further characterize the spatiotemporal dynamics of ZIKV following introduction into this region [26]. In addition to sequencing data, air traffic data for visitors to Brazil from other countries associated with major social events during 2012–2014 were included to test different hypotheses of airline-mediated introduction of ZIKV in Brazil. The results linked the origin of the Brazilian epidemic to a single introduction of ZIKV estimated to occur between May and December 2013, consistent with the Confederations Cup event, but predating the first reported cases in French Polynesia. Although these findings are of great value and importance to public health organizations, the authors drew an additional, and similarly valuable conclusion–large-scale patterns in human (and mosquito) mobility extending beyond air traffic data will provide more useful and testable hypotheses about disease emergence and spread than ad hoc hypotheses focused on specific events. This conclusion further supports the proposal for greater availability of epidemiological data among the scientific community.

Understanding both the rapid spread of the virus throughout South and Central America and the Caribbean as well as the initial emergence of the virus from the Ugandan Zika forest in the early 1900s is important for application to the control of future outbreaks, but increasing data may not be the only answer. Moreover, several different risk factors are likely responsible for these two migration events. Therefore, a more comprehensive approach that allows for the analysis of multiple potential factors and their distinct contribution to independent migration events without the loss of information (i.e., use of data that span the entire evolutionary history) is imperative for fully understanding a global epidemic from beginning to present.

## A combined approach to understanding the emergence and expansion of an epidemiologically diverse viral population: HIV CRF02_AG in the Congo River basin

Although viral spread is often attributed to human mobility [71], factors such as population growth and accessibility can also play an important role, as with the emergence of human immunodeficiency virus type 1 (HIV-1) group M subtypes A and D in east Africa [72] and circulating recombinant form (CRF) 02_AG in regions of the Congo River basin (CRB) [73]. The democratic republic of Congo (DRC) has been reported to be the source of HIV-1 group M diversity [74–76]; however, the epidemiological heterogeneity of CRF02_AG within surrounding regions comprising the CRB had remained a mystery since its discovery in 1994 [77], with prevalence ranging from virtual non-existence [78–83] to accounting for as high as 20% of infections [84], depending on the geographical location. The region with the highest proportion of CRF02_AG infections, Cameroon [85, 86], has been characterized by a rapidly growing infected population (0.5% in 1985 to 6% in 2008 [87]), of which the majority (60%) is caused by this clade. Using both molecular sequence data and UNAIDS surveillance data [88], the spatiotemporal origin of CRF02_AG was estimated to occur in the DRC in the early 1970s (1972–1975), with the rapid viral population growth in Cameroon following a chance exportation event out of DRC.

Although similar phylodynamic techniques as described above for other viral species were used to infer the spatial origins of CRF02_AG, the timing of the origin of this viral clade was inferred using both coalescent analysis of molecular sequence data and prevalence information [73, 89]. Coalescent models allow for estimation of the effective population size (*Ne*), of fundamental importance to infectious disease epidemiology, as it describes the level of genetic diversity within a population over the course of its evolutionary history. During the exponential growth period of an epidemic, the change in *Ne* has been shown to linearly correlate with prevalence of infection [90, 91] and can, therefore, be used to estimate the latter, as mentioned above, but also, when combined, Faria et al. [73] were able to show that fitting of *Ne* and prior prevalence data can narrow the uncertainty of the temporal origin estimates by over 29% as compared to coalescent estimates alone. Furthermore, surveillance data was recently used during simultaneous phylodynamic coalescent estimation to identify factors associated with *Ne* dynamics throughout the entire evolutionary history of the Cameroonian sequences [92], revealing that changes in *Ne* were more reflective of incidence dynamics rather than prevalence, consistent with previous mathematical modeling [90, 91]. Although associations between *Ne* and potentially related factors are frequently assessed, statistical analysis of these has until recently been primarily limited to *post hoc* examination (e.g., [91, 93]), which ignores uncertainty in demographic reconstruction, as discussed above. Simultaneous implementation of evolutionary reconstruction and estimation of the relationship of covariate data with *Ne* will be available in the newest version of BEAST v1 [92]. Although this tool has obvious implications for global assessment of factors contributing to the growth and dynamics of an epidemic, similar applications of this method to other data sets has suggested that reduced molecular data relative to covariate data may result in an impact of inclusion of the data on *Ne* estimates. This finding posits a potential concern for convenience sequence sampling, as factors that are not responsible but are represented by large amounts of data may influence *Ne* estimates, resulting in unreliable population dynamic inferences. As mentioned above, care is needed to ensure sufficient sampling and an appropriate sampling strategy for reliable reconstruction of the evolutionary and epidemiological history of the infectious organism of interest.

## Tracing the source of nosocomial outbreaks: Methicillin-resistant *Staphylococcus aureus*

Traditional phylodynamic analysis applied to nosocomial outbreaks has been successfully used in the past to identify the likely source; however, the inclusion of extensive patient data, such as treatment regimens, admission and discharge dates, and length of stay, can improve not only phylogenetic estimates but also the translation of the interpretation to public health policy. Epidemiological and genomic data on Methicillin-resistant *Staphylococcus aureus* (MRSA) infections were recently utilized by Azarian and colleagues to reconstruct MRSA transmission and to estimate possible community and hospital acquisitions [94]. Findings from this study revealed that as high as 70% of the MRSA colonization within the hospital’s neonatal intensive care unit (NICU) was acquired within the NICU itself. These findings indicated that current, standard prevention efforts were insufficient in preventing an outbreak, calling for the improvement of current care or alternative implementation strategies.

## Conclusions

The earlier uses of phylodynamic methods focused primarily on the molecular evolution of rapidly evolving viruses, greatly advancing the fields of virus vaccine and treatment strategies [23]. On the other hand, epidemiological approaches have focused on influential factors related to social, economic, and behavioral patterns. Integrating the phylodynamics and epidemiology approaches into a single analytical framework, referred to as evolutionary epidemiology [24, 25], represents one of the most powerful multi-disciplinary platforms. Examples discussed herein of the adoption of an integrative and multifactorial mindset reveal the potential for accelerating our understanding of the emergence and spread of global infectious diseases, presently expanded to include bacterial and other cell-based pathogens. However, although a highly evolved analytical platform and an improved understanding of the translation of molecular evolutionary patterns to infection and transmission dynamics have aided in facilitating this transition, several challenges still remain.

The 21^st^ century has witnessed a major shift in breadth of scientific knowledge at the level of the individual researcher, requiring more focused training (e.g., molecular mechanisms) and greater collaborative efforts; meanwhile, a consensus of commonality and cross-disciplinary understanding is necessary for globalization of not only the economy, but also public health. This kind of understanding can be better achieved through interdisciplinary instruction on the theoretical and application skills related to both phylogenetics and epidemiology during early education. If successfully achieved, this combined training, in addition to access to modern NGS technology, such as handheld sequencers, would increase the mobility of labs and researchers, expanding the concept of lab-based research. Mobilized labs would, in turn, reduce our current reliance on few major public health organizations and the impact of limited resources on sampling and surveillance in developing countries.

Increasing mobility is nevertheless inconsequential without the cooperative sharing of genomic and epidemiological information. Although data are typically readily available to the public following peer-reviewed publication, the median review time of manuscripts submitted to, for example, *Nature* is 150 days [95], this in addition to the time required for thorough analysis of the original data. This timeline seems quite long in retrospect of the 1918 “Spanish flu,” which spread to one-third of the global population in a relatively brief 12-month period [96]. Data sharing prior to publication, even if only among a proportion of consenting institutions, may accelerate the process of dissemination of research findings to public health decision makers and practitioners, and its practice is not entirely unheard of. An excellent example of this type of collaboration is the “nextstrain” project (http://www.nextstrain.org/). Nextstrain is a publicly available repository currently comprised of evolutionary datasets for Ebola, Zika, and avian and seasonal influenza viruses contributed by research groups from all over the world for the purpose of real-time tracking of viral epidemics. Similar projects have also recently developed in other research fields. Modeled after the Stand up to Cancer initiative, the Synodos collaborative funded by the Children’s Tumor Foundation in partnership with Sage Bionetworks brings together a consortium of multidisciplinary researchers, who have agreed to the sharing of data and relevant information, as well as results [97]. The ultimate goal of this cooperation is to accelerate the drug discovery process, which is highly applicable to global infectious disease research.

Without a similar collaborative approach to Synodos, the preparedness of the global reaction to rising epidemics is at risk. Recent years have been marked by local outbreaks across vast geographical regions within a timespan of months to years. Hence, both the rapid dissemination of data and results and the rapid response of government and public health organizations are required for the effective prevention of a global epidemic, or pandemic. Additionally, with the type of results, particularly risk factors, that are generated using this multifaceted approach (e.g., both human population and pathogen molecular characteristics), the question then arises of how organizations will actually utilize this information for treatment and prevention strategies. Moreover, as the techniques and methods advance, are the infrastructures in place for global cooperation and immediate response following the presentation of a potentially more complex story?

Although gaps remain in current evolutionary modeling capabilities when used with epidemiological surveillance data, it is only a matter of time before the challenges described herein and elsewhere are met with more realistic models that capture the complexity of infectious disease transmission. Furthermore, theoretical research in the field of infectious disease phylodynamics is still growing. Consequently, there is a need for a review of the more recently developed methods and techniques and their performance, as well as their application in areas within and outside the realm of infectious disease. For example, in the era of global health, translational genomics, and personalized medicine, the accumulating availability of genetic and clinical data provides the unique opportunity to apply this approach to studies of, e.g., tumor metastasis and chronic infections, which comprise complex transmission dynamics among tissues and/or cell types, not unlike the geographical spread of infectious diseases.

## Abbreviations

BEAST: Bayesian Evolutionary Analysis Sampling Trees
DENV: Dengue virus
GIS: Geographical information systems
HIV: Human immunodeficiency virus
MRSA: Methicillin-resistant *Staphylococcus aureus*
NGS: Next-generation sequencing
NICU: Neonatal intensive care unit
SNP: Nucleotide polymorphism
WGS: Whole-genome sequencing
ZIKV: Zika virus

## References

[1] Lee K, Yach D, Kamradt-Scott A (2011). Globalization and Health.

[2] Chen X (2014). Understanding the development and perception of global health for more effective student education. Yale J Biol Med.

[3] Gushulak BD, MacPherson DW (2004). Globalization of infectious diseases: the impact of migration. Clin Infect Dis.

[4] Lafferty KD (2009). The ecology of climate change and infectious diseases. Ecology.

[5] Patz J, Epstein PR, Burke T (1996). Global climate change and emerging infectious diseases. J Am Med Assoc.

[6] Tambo E, Chuisseu PD, Ngogang JY (2016). Deciphering emerging Zika and dengue viral epidemics: Implications for global maternal–child health burden. J Infect Public Health.

[7] World Health Organization (2016). World Health Statistics: 2016.

[8] Abat C, Chaudet H, Rolain J-M (2016). Traditional and syndromic surveillance of infectious diseases and pathogens. Int J Infect Dis.

[9] Hernán MA, Hernández-Díaz S, Robins JM (2004). A Structural Approach to Selection Bias. Epidemiology.

[10] Coughlin SS (1990). Recall bias in epidemiologic studies. J Clin Epidemiol.

[11] Furnham A (1986). Response bias, social desirability and dissimulation. Personal Individ Differ.

[12] De Luca A, Prosperi M, Bracciale L (2010). Resistance considerations in sequencing of antiretroviral therapy in low-middle income countries with currently available options. Curr Opin HIV AIDS.

[13] Vernet G, Mary C, Altmann DM (2014). Surveillance for Antimicrobial Drug Resistance in Under-Resourced Countries. Emerg Infect Dis.

[14] Buchbinder SP, Liu AY (2013). CROI 2013: New tools to understand transmission dynamics and prevent HIV infections. Top Antivir Med.

[15] Gebreyes WA, Dupouy-Camet J, Newport MJ, et al. The Global One Health Paradigm: Challenges and Opportunities for Tackling Infectious Diseases at the Human, Animal, and Environment Interface in Low-Resource Settings. PLoS Negl Trop Dis. 2014;8. doi:10.1371/journal.pntd.0003257. 10.1371/journal.pntd.0003257 PMC4230840 25393303

[16] Brownson RC, Samet JM, Chavez GF (2015). Charting a future for epidemiologic training. Ann Epidemiol.

[17] Kuller LH, Bracken MB, Ogino S (2013). The role of epidemiology in the era of molecular epidemiology and genomics: Summary of the 2013 AJE-sponsored Society of Epidemiologic Research Symposium. Am J Epidemiol.

[18] Khoury MJ, Lam TK, Ioannidis JPAA (2013). Transforming Epidemiology for 21st Century Medicine and Public Health. Cancer Epidemiol Biomarkers Prev.

[19] March D, Susser E (2006). The eco- in eco-epidemiology. Int J Epidemiol.

[20] Susser E (2004). Eco-Epidemiology: Thinking Outside the Black Box. Epidemiology.

[21] Volz EEM, Koelle K, Bedford T (2013). Viral phylodynamics. PLoS Comput Biol.

[22] Henning KJ (2004). Overview of Syndromic Surveillance What is Syndromic Surveillance?. MMWR.

[23] Grenfell BT, Pybus OG, Gog JR (2004). Unifying the Epidemiological and Evolutionary Dynamics of Pathogens. Science.

[24] Kühnert D, Wu C-H, Drummond AJ (2011). Phylogenetic and epidemic modeling of rapidly evolving infectious diseases. Infect Genet Evol.

[25] Pybus OG, Fraser C, Rambaut A (2013). Evolutionary epidemiology: preparing for an age of genomic plenty. Philisophical Trans R Soc B Biol Sci.

[26] Faria NR, da Silva Azevedo dS, Kraemer MUG, et al. Zika virus in the Americas: Early epidemiological and genetic findings. Science. 2016;352:345–9. doi:10.1126/science.aaf5036. 10.1126/science.aaf5036 PMC4918795 27013429

[27] Lam TT-Y, Zhu H, Guan Y (2016). Genomic Analysis of the Emergence, Evolution, and Spread of Human Respiratory RNA Viruses. Annu Rev Genomics Hum Genet.

[28] Park DJ, Dudas G, Wohl S (2015). Ebola Virus Epidemiology, Transmission, and Evolution during Seven Months in Sierra Leone. Cell.

[29] Bos KI, Herbig A, Sahl J (2016). Eighteenth century *Yersinia pestis* genomes reveal the long-term persistence of an historical plague focus. Elife.

[30] Min J, Cella E, Ciccozzi M (2016). The global spread of Middle East respiratory syndrome: an analysis fusing traditional epidemiological tracing and molecular phylodynamics. Glob Health Res Policy.

[31] Haeckel E (1876). History of Creation.

[32] Hall MD, Woolhouse MEJ, Rambaut A (2016). The effects of sampling strategy on the quality of reconstruction of viral population dynamics using Bayesian skyline family coalescent methods: A simulation study. Virus Evol.

[33] Rambaut A, Lam TT, Carvalho LM (2016). Exploring the temporal structure of heterochronous sequences using TempEst (formerly Path-O-Gen). Virus Evol.

[34] Norström MM, Karlsson AC, Salemi M (2012). Towards a new paradigm linking virus molecular evolution and pathogenesis: experimental design and phylodynamic inference. New Microbiol.

[35] Lemmon AR, Brown JM, Stanger-Hall K (2009). The Effect of Ambiguous Data on Phylogenetic Estimates Obtained by Maximum Likelihood and Bayesian Inference. Syst Biol.

[36] Martin DP, Lemey P, Posada D (2011). Analysing recombination in nucleotide sequences. Mol Ecol Resour.

[37] Huson DH, Bryant D (2006). Application of phylogenetic networks in evolutionary studies. Mol Biol Evol.

[38] Woolley SM, Posada D, Crandall KA (2008). A Comparison of Phylogenetic Network Methods Using Computer Simulation. PLoS One.

[39] Faria NR, Rambaut A, Suchard MA (2014). The early spread and epidemic ignition of HIV-1 in human populations. Science.

[40] Simon-Loriere E, Holmes EC (2011). Why do RNA viruses recombine?. Nat Rev Microbiol.

[41] Gill MS, Lemey P, Faria NR (2013). Improving Bayesian population dynamics inference: a coalescent-based model for multiple loci. Mol Biol Evol.

[42] Biek R, Pybus OG, Lloyd-Smith JO (2015). Measurably evolving pathogens in the genomic era. Trends Ecol Evol.

[43] Fraser C, Donnelly CA, Cauchemez S (2009). Pandemic potential of a strain of influenza A (H1N1): early findings. Science.

[44] Heller R, Chikhi L, Siegismund HR (2013). The Confounding Effect of Population Structure on Bayesian Skyline Plot Inferences of Demographic History. PLoS One.

[45] Drummond AJ, Suchard MA, Xie D (2012). Bayesian phylogenetics with BEAUti and the BEAST 1.7. Mol Biol Evol.

[46] Drummond A, Rambaut A (2007). BEAST: Bayesian evolutionary analysis by sampling trees. BMC Evol Biol.

[47] Bouckaert R, Heled J, Kühnert D (2014). BEAST 2: A Software Platform for Bayesian Evolutionary Analysis. PLoS Comput Biol.

[48] Lemey P, Rambaut A, Drummond AJ (2009). Bayesian Phylogeography Finds Its Roots. PLoS Comput Biol.

[49] Minin VN, Suchard MA (2007). Counting labeled transitions in continuous-time Markov models of evolution. J Math Biol.

[50] Minin VN, Suchard MA (2008). Fast, accurate and simulation-free stochastic mapping. Philos Trans R Soc Lond Ser B Biol Sci.

[51] Rasmussen DA, Volz EM, Koelle K (2014). Phylodynamic Inference for Structured Epidemiological Models. PLoS Comput Biol.

[52] De Maio N, Wu C-H, O’Reilly KM (2015). New Routes to Phylogeography: A Bayesian Structured Coalescent Approximation. PLoS Genet.

[53] Vaughan TG, Kühnert D, Popinga A (2014). Efficient Bayesian inference under the structured coalescent. Bioinformatics.

[54] Vrancken B, Lemey P, Rambaut A (2015). Simultaneously estimating evolutionary history and repeated traits phylogenetic signal: applications to viral and host phenotypic evolution. Methods Ecol Evol.

[55] Beard R, Magee D, Suchard MA (2014). Generalized linear models for identifying predictors of the evolutionary diffusion of viruses. AMIA Jt Summits Transl Sci Proc AMIA Jt Summits Transl Sci.

[56] Kolaczkowski B, Thornton JW, Hillis D (2009). Long-Branch Attraction Bias and Inconsistency in Bayesian Phylogenetics. PLoS One.

[57] Susko E (2008). On the Distributions of Bootstrap Support and Posterior Distributions for a Star Tree. Syst Biol.

[58] Nguyen L-T, Schmidt HA, von Haeseler A (2015). IQ-TREE: A Fast and Effective Stochastic Algorithm for Estimating Maximum-Likelihood Phylogenies. Mol Biol Evol.

[59] Münkemüller T, Lavergne S, Bzeznik B (2012). How to measure and test phylogenetic signal. Methods Ecol Evol.

[60] Kapoor A, Lipkin WI, Kapoor A (2014). Virus Discovery in the 21st Century. eLS.

[61] Baele G, Lemey P (2013). Bayesian evolutionary model testing in the phylogenomics era: matching model complexity with computational efficiency. Bioinformatics.

[62] Berger J (2004). The Case for Objective Bayesian Analysis. Bayesian Anal.

[63] van de Schoot R, Broere JJ, Perryck KH, et al. Analyzing small data sets using Bayesian estimation: the case of posttraumatic stress symptoms following mechanical ventilation in burn survivors. Eur J Psychotraumatol. 2015;6. doi:10.3402/ejpt.v6.25216. 10.3402/ejpt.v6.25216 PMC4357639 25765534

[64] Chretien J-P, Rivers CM, Johansson MA (2016). Make Data Sharing Routine to Prepare for Public Health Emergencies. PLoS Med.

[65] Capua I (2016). A code of conduct for data on epidemics. Nature.

[66] Lemey P, Rambaut A, Bedford T (2014). Unifying viral genetics and human transportation data to predict the global transmission dynamics of human influenza H3N2. PLoS Pathog.

[67] Nunes MRT, Palacios G, Faria NR (2014). Air travel is associated with intracontinental spread of dengue virus serotypes 1-3 in Brazil. PLoS Negl Trop Dis.

[68] dos Reis M, Donoghue PCJ, Yang Z (2015). Bayesian molecular clock dating of species divergences in the genomics era. Nat Rev Genet.

[69] Kindhauser MK, Allen T, Frank V (2016). Zika: the origin and spread of a mosquito-borne virus. Bull World Health Organ.

[70] Faye OO, Freire CCM, Iamarino A (2014). Molecular Evolution of Zika Virus during Its Emergence in the 20th Century. PLoS Negl Trop Dis.

[71] Quinn TC (1994). Population migration and the spread of types 1 and 2 human immunodeficiency viruses. Proc Natl Acad Sci U S A.

[72] Gray RR, Tatem AJ, Lamers S (2009). Spatial phylodynamics of HIV-1 epidemic emergence in east Africa. AIDS.

[73] Faria NR, Suchard MA, Abecasis A (2012). Phylodynamics of the HIV-1 CRF02_AG clade in Cameroon. Infect Genet Evol.

[74] Sharp PM, Hahn BH (2008). AIDS: Prehistory of HIV-1. Nature.

[75] Rambaut A, Robertson DL, Pybus OG (2001). Human immunodeficiency virus: Phylogeny and the origin of HIV-1. Nature.

[76] Vidal N, Peeters M, Mulanga-Kabeya C (2000). Unprecedented degree of human immunodeficiency virus type 1 (HIV-1) group M genetic diversity in the Democratic Republic of Congo suggests that the HIV-1 pandemic originated in Central Africa. J Virol.

[77] Howard TM, Olaylele DO, Rasheed S (1994). Sequence Analysis of the Glycoprotein 120 Coding Region of a New HIV Type 1 Subtype A Strain (HIV-1 IbNg) from Nigeria. AIDS Res Hum Retroviruses.

[78] Kita K, Ndembi N, Ekwalanga M (2004). Genetic Diversity of HIV Type 1 in Likasi, Southeast of the Democratic Republic of Congo. AIDS Res Hum Retroviruses.

[79] Bikandou B, Takehisa J, Mboudjeka I (2000). Genetic Subtypes of HIV Type 1 in Republic of Congo. AIDS Res Hum Retroviruses.

[80] Niama FR, Toure-Kane C, Vidal N (2006). HIV-1 subtypes and recombinants in the Republic of Congo. Infect Genet Evol.

[81] Marechal V, Jauvin V, Selekon B (2006). Increasing HIV Type 1 Polymorphic Diversity But No Resistance to Antiretroviral Drugs in Untreated Patients from Central African Republic: A 2005 Study. AIDS Res Hum Retroviruses.

[82] Müller-Trutwin MC, Chaix ML, Letourneur F (1999). Increase of HIV-1 subtype A in Central African Republic. J Acquir Immune Defic Syndr.

[83] Bártolo I, Rocha C, Bartolomeu J (2009). Highly divergent subtypes and new recombinant forms prevail in the HIV/AIDS epidemic in Angola: New insights into the origins of the AIDS pandemic. Infect Genet Evol.

[84] Pandrea I, Robertson DL, Onanga R (2002). Analysis of Partial *pol* and *env* Sequences Indicates a High Prevalence of HIV Type 1 Recombinant Strains Circulating in Gabon. AIDS Res Hum Retroviruses.

[85] Brennan CA, Bodelle P, Coffey R (2008). The Prevalence of Diverse HIV-1 Strains Was Stable in Cameroonian Blood Donors From 1996 to 2004. JAIDS J Acquir Immune Defic Syndr.

[86] Carr JK, Osinusi A, Flynn CP (2010). Two Independent Epidemics of HIV in Maryland. JAIDS J Acquir Immune Defic Syndr.

[87] World Health Organization. WHO | Epidemiological fact sheets on HIV and AIDS, 2008 update. WHO; 2009. http://www.who.int/hiv/pub/epidemiology/pubfacts/en/. Accessed 29 Mar 2017.

[88] UNAIDS. AIDSinfo. http://aidsinfo.unaids.org/. Accessed 29 Mar 2017.

[89] Bennett SN, Drummond AJ, Kapan DD (2010). Epidemic Dynamics Revealed in Dengue Evolution. Mol Biol Evol.

[90] Frost SDW, Volz EM (2010). Viral phylodynamics and the search for an ‘effective number of infections’. Philos Trans R Soc Lond Ser B Biol Sci.

[91] Volz EM, Kosakovsky Pond SL, Ward MJ (2009). Phylodynamics of Infectious Disease Epidemics. Genetics.

[92] Gill MS, Lemey P, Bennett SN (2016). Understanding Past Population Dynamics: Bayesian Coalescent-Based Modeling with Covariates. Syst Biol.

[93] Biek R, Henderson JC, Waller LA (2007). A high-resolution genetic signature of demographic and spatial expansion in epizootic rabies virus. Proc Natl Acad Sci.

[94] Azarian T, Maraqa NF, Cook RL (2016). Genomic Epidemiology of Methicillin-Resistant Staphylococcus aureus in a Neonatal Intensive Care Unit. PLoS One.

[95] Powell K (2016). Does it take too long to publish research?. Nature.

[96] Taubenberger JK, Morens DM (2006). 1918 Influenza: the Mother of All Pandemics. Emerg Infect Dis.

[97] Dream Team of Scientists Collaborate in Unique NF Research Consortium. Children’s Tumor Foundation Announces Historic New Initiative in Neurofibromatosis Research; 2014. https://globenewswire.com/news-release/2014/03/10/617113/10071956/en/Children-s-Tumor-Foundation-Announces-Historic-New-Initiative-in-Neurofibromatosis-Research.html. Accessed 12 Dec 2016.

